# Mechanical and Anticorrosive Properties of Graphene/Epoxy Resin Composites Coating Prepared by *in-Situ* Method

**DOI:** 10.3390/ijms16012239

**Published:** 2015-01-20

**Authors:** Zhiyi Zhang, Wenhui Zhang, Diansen Li, Youyi Sun, Zhuo Wang, Chunling Hou, Lu Chen, Yang Cao, Yaqing Liu

**Affiliations:** 1 Research Center for Engineering Technology of Polymeric Composites of Shanxi Province, North University of China, Taiyuan 030051, China; E-Mails: zhangY@163.com (Z.Z.); WangZ@163.com (W.Z.); syyi2010@163.com (Z.W.); houchunlin0329@163.com (C.H.); caoyang800929@126.com (Y.C.); 2 Key Laboratory of Bio-Inspired Smart Interfacial Science and Technology of Ministry of Education, Beijing Key Laboratory of Bio-Inspired Energy Materials and Devices, School of Chemistry and Environment, Beijing University of Aeronautics and Astronautics, Beijing 100191, China; E-Mail: LiDS@163.com; 3 College of Resources and Environmental Science, Nanjing Agricultural University, Nanjing 210095, China; E-Mail: Chenglu@163.com

**Keywords:** graphene, epoxy resin, composite coating, mechanical properties, erosion resistance

## Abstract

The graphene nanosheets-based epoxy resin coating (0, 0.1, 0.4 and 0.7 wt %) was prepared by a situ-synthesis method. The effect of polyvinylpyrrolidone/reduced graphene oxide (PVP-rGO) on mechanical and thermal properties of epoxy resin coating was investigated using nanoindentation technique and thermogravimetric analysis, respectively. A significant enhancement (*ca.* 213% and 73 °C) in the Young modulus and thermal stability of epoxy resin coating was obtained at a loading of 0.7 wt %, respectively. Furthermore, the erosion resistance of graphene nanosheets-based epoxy resin coating was investigated by electrochemical measurement. The results showed also that the Rrcco (*ca.* 0.3 mm/year) of graphene nanosheets-based epoxy resin coating was far lower than neat epoxy resin (1.3 mm/year). Thus, this approach provides a novel route for improving erosion resistance and mechanical-thermal stability of polymers coating, which is expected to be used in mechanical-thermal-corrosion coupling environments.

## 1. Introduction

Epoxy resin is the most common polymeric coating that inhibited the process of metal corrosion due to high tensile strength and modulus, low shrinkage in cure, good chemical and corrosion resistance, high adhesion and dimensional stability [1]. On the other hand, it has been experimentally demonstrated that the graphene coating effectively suppresses metal oxidation by oxygen reduction and metal salt solution. Most importantly, graphene sheets can protect the polymer underneath from atomic oxygen (AO) erosion because they pose a high energy barrier to AO diffusing from the top of the graphene sheets to the reactive polymer surface underneath. These results indicate the functional capabilities of graphene as effective corrosion-inhibiting materials [2,3]. So, graphene nanosheets (GNS)-based epoxy resin coating is expected to be a good kind of anticorrosive material. Although epoxy resin/graphene nanocomposites have attracted a tremendous amount of attention because graphene fillers with very low loading have the potential to match or exceed the performance of large quantities of traditional composite fillers, most works have mainly emphasized enhancing mechanical, electrical or flame retardancy properties of epoxy resin by adding graphene fillers [4,5,6,7,8,9,10,11]. Recently, the anticorrosive properties of GNS-based epoxy resin coating were reported [12], in which enhancements of corrosion protection using GNS-based epoxy resin coatings were observed. It is well-known that polymeric coatings are generally used in mechanical-thermal-corrosion coupling environments. So, the mechanical and thermal stability of polymeric coatings is also a key factor for their application in industry. For example, when the coating barrier is mechanically or thermally damaged and the corrosive species penetrate the metal surface, the corrosion process cannot be avoided [13,14]. So, the study in mechanical and thermal properties of polymer coating is very important for developing anticorrosive properties. However, few mechanical and thermal properties (*i.e*., Young’s modulus, hardness and plastic index) of polymer coatings have been characterized and reported.

In this study, the GNS-based epoxy resin coating was prepared by the *situ*-synthesis method. Furthermore, nanoindentation technique was used to measure the mechanical properties of GNS-based epoxy resin coating, in which the Young’s modulus, hardness and plastic index could be obtained. Furthermore, erosion resistance of GNS-based epoxy resin coating was also determined by electrochemical measurements. These results are very important for improving the erosion resistance of polymer coating, which is expected to be used in multi-field coupling environments.

## 2. Results and Discussion

### 2.1. Preparation of GNS-Based Epoxy Resin Coating

**Figure 1:**
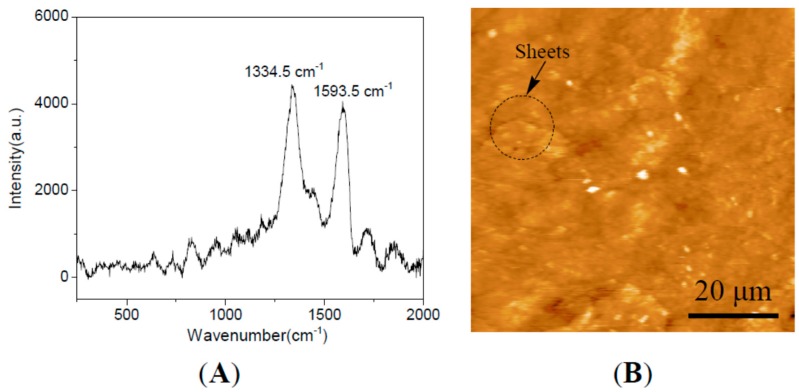
(**A**) Raman spectra; and (**B**) AFM of GNS-based epoxy resin coating.

The typical Raman spectra of GNS-based epoxy resin coating were shown in Figure 1A. The integrated intensity over the spectral ranges 1250~1450 and 1500~1700 cm^−1^ were attributed to the location of the carbon-related D peak and the location of the carbon-related graphene peak, indicating the dispersion of the graphene in the epoxy resin matrix [15]. The GNS-based epoxy resin coating was further characterized by the atomic force microscopic (AFM) measurements as shown in Figure 1B. It clearly shows some micrometer-sized sheets in epoxy resin matrix, which were assigned to graphene sheets. At the same time, it also clearly shows a smooth surface and no aggregates for the polymer coating. These results confirm the formation of GNS-based epoxy resin coating, in which the graphene sheet demonstrates good dispersion in the epoxy resin matrix.

**Figure 2:**
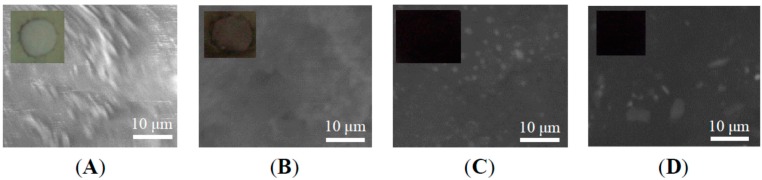
SEM of GNS-based epoxy resin coating with various content of (**A**) 0; (**B**) 0.1 wt %; (**C**) 0.4 wt %; and (**D**) 0.7 wt %. The corresponding digital photographs of GNS-based epoxy resin coating are shown in the inset.

The inset of Figure 2 showed the digital photographs of neat epoxy resin coating and GNS-based epoxy resin coating. One could see that, with incorporation of only 0.1 wt % graphene, the transparent epoxy resin coating became rather dark, indicating the uniform dispersion of graphene sheets in the epoxy resin matrix. With the increase of the doping content, the color of the GNS-based epoxy resin coating became darker and darker. At the same time, the surfaces of GNS-based epoxy resin coating displayed a generally smooth morphology, indicating that the epoxy resin coating blending with graphene were miscible. The GNS-based epoxy resin coating was also characterized by the SEM as shown in Figure 2, too. They clearly showed some micrometer-sized sheets in epoxy resin matrix, in which the averages size of sheets was about 2 μm. In addition to this, it also clearly showed that there were no agglomerate and gaps between graphene and epoxy resin. These results further indicate that the PVP-rGO can be well dispersed in the epoxy resin matrix and have good adhesion with epoxy resin matrix.

### 2.2. Mechanical Properties of GNS-Based Epoxy Resin Coating

#### 2.2.1. Nanoindentation Behavior

In the present research, the nanoindentation test was used to measuring the elastic modulus (*E*) and the hardness (*H*) of GNS-based epoxy resin coating [16]. They showed that the hardness and the elastic modulus could be measured by analyzing the unloading part of the load displacement curve. The indentation hardness is given by:

(1) $$H=\frac{P_{\max}}{A}$$

where, *P*_max_ is the maximum normal load and *A* is the projected contact area at the maximum load. The elastic modulus (*E*) can be calculated from the following equations:

(2) $$\begin{array}{l} E_{r}=\frac{\sqrt{\text{π}}}{2}\frac{dp}{dh}\frac{1}{\sqrt{A}}\\ \frac{1}{E_{r}}=\frac{1-{\text{ν}}^{2}}{E}+\frac{1-{\text{ν}}_{i}^{2}}{E_{i}} \end{array}$$

where, *E_r_* is the reduced modulus of indentation contact, *E_i_* (1140 GPa) and ν_i_ (0.07) are the elastic modulus and Poisson’s ratio of the indenter, and ν = 0.40 is the Poisson’s ratio of the test sample [17].

**Figure 3:**
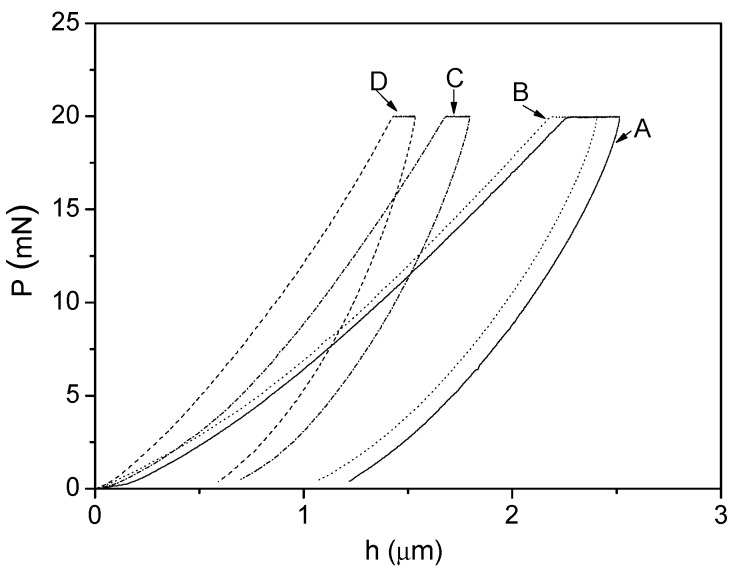
Load-displacement curves of GNS-based epoxy resin coating with different contents of (A) 0; (B) 0.1 wt %; (C) 0.4 wt %; and (D) 0.7 wt %.

Figure 3 shows loading-unloading curves of neat epoxy resin coating and GNS-based epoxy resin coating. These curves were obtained from the nanoindentation test with a normal force of 800 mN. By increasing PVP-rGO content, nanoindentation curves shifted to the left and maximum depth reduced due to the increasing hardness. The Young’s modulus and hardness was concluded in Table 1. It clearly showed that modulus and hardness of GNS-based epoxy resin coating increased with increases in PVP-rGO content. For instance, the hardness and elastic modulus of GNS-based epoxy resin coating (0.7 wt %) was improved equaling 294% and 213% compared with that of neat epoxy resin coating, respectively. These results were attributed to the fact that graphene is the strongest material known to man [18,19]. Modulus and stiffness have a direct relationship and growth in stiffness will result in a higher modulus. Hardness has been defined as the resistance of material surface against the deformation caused by normal force, and elastic modulus is the ability of the material to recover its former shape. The indenter interacted with more graphene sheets by increasing content of PVP-rGO. So, the material resists more against the deformation caused by normal load and shows better elastic recovery. Consequently, hardness and modulus showed huge improvement with increases in PVP-rGO content. However, the huge improvement of hardness and elastic modulus (*i.e.*, 294% and 213%) was firstly reported [6,7,8,9,10,11,20]. The good dispersion and adhesion of PVP-rGO in epoxy resin matrix also played an important role.

**Table 1:** Mechanical properties of GNS-based epoxy resin coating.

Loading	Er (GPa)	Young’s Modulus (GPa)	Hardness (GPa)	Plasticity Index (%)
0	2.90	2.44	0.17	43.4
0.1%	3.10	2.60	0.19	43.1
0.4%	4.33	3.64	0.41	34.4
0.7%	6.69	5.62	0.51	31.7

#### 2.2.2. Plasticity Index

The plasticity index (ψ) is usually used to characterize the elastic-plastic response of a material under external stresses and strains. In the nanoindentation test, the plasticity index of the material can be calculated as follows [16]:

(3) $$\Psi =\frac{A_{1}-A_{2}}{A_{1}}$$

where *A*_1_ is the area under the loading curve and *A*_2_ is the area under the unloading curve (see Figure 3). The area below the loading curve equals to the total spent energy during the indentation and the area below the unloading curve equals energy released during unloading. The difference between these two areas indicates the irreversible work during the nanoindentation test. For materials with viscoelastic-plastic behavior such as polymers, the plasticity index is in the range of 0 < ψ < 1. Additionally, ψ = 0 and ψ = 1 representing the fully-elastic and the fully-plastic behavior of materials, respectively.

Table 1 also showed the values of plasticity index for neat epoxy resin coating and GNS-based epoxy resin coating. By increasing the content of PVP-rGO, the plasticity index showed a reduction, indicating the improvement in elastic recovery of GNS-based epoxy resin coating. The reduction of this parameter at different PVP-rGO contents (*i.e*., 0.1, 0.4 and 0.7 wt %) was 0.7%, 20.5% and 26.9%, respectively. At higher contents of PVP-rGO, polymer chains in contact with PVP-rGO have less time to recover elastically after removing the external load. Hence, there was more plastic deformation remaining. At the same time, it was found that the plasticity index of GNS-based epoxy resin coating was lower than that reported in previous works [16,21]. The result was attributed to good adhesion and dispersion of graphene modified by PVP in epoxy resin matrix.

**Figure 4:**
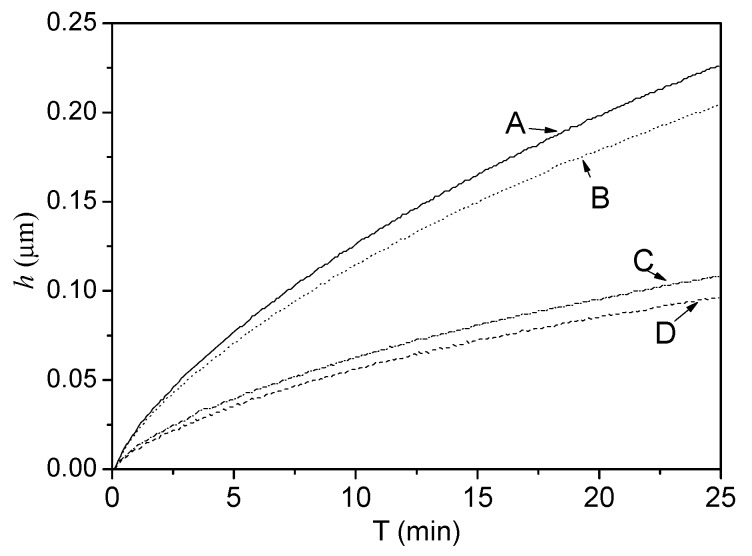
Load-displacement curves of GNS-based epoxy resin coating with different contents of (A) 0; (B) 0.1 wt %; (C) 0.4 wt %; and (D) 0.7 wt %.

The nanoindentation creep of GNS-based epoxy resin coating was further determined as shown in Figure 4, in which the strain (*h*) was sensitivity to hold time. As shown in Figure 4, the strain (*h*) increased with increasing in hold time, indicating the presence of creep for all samples. At the same time, it was found that the creep rate of GNS-based epoxy resin coating decreased with increasing in content of PVP-rGO. For example, by adding 0.7 wt % PVP-rGO, it always resulted in a smaller creep rate sensitivity parameter (0.23 μm/h) compared to that (0.54 μm/h) of the neat epoxy resin coating. The ability of the PVP-rGO additives to increase creep resistance, manifested by the creep lifetime prolongation and creep rate reduction, could be attributed to following several factors: the confinement effect of the highly elastic PVP-rGO prevented the deformation of the epoxy network; the PVP-rGO acted as sites, blocking the movement of the epoxy chains when subjected to an external indentation force, hence, they restricted the viscous flow of the amorphous epoxy; Regardless of the nature of the loading (tensile, shear,* etc.*), ensuring a good load transfer between the epoxy matrix and the PVP-rGO may give rise to good creep resistance of the GNS-based epoxy resin as a whole. The enhancement in creep resistance can also be related to several nano effects of the PVP-rGO such as their high aspect ratio and large interfacial area which contributes to significant interface adhesion between the PVP-rGO and the epoxy matrix and thus improves the load transfer. This network interconnects the epoxy chains, thus increases the cross linking density and restricts the mobility of the epoxy chains under nanoindentation mechanical loading [22,23,24].

### 2.3. Thermal Properties of GNS-Based Epoxy Resin Coating

**Figure 5:**
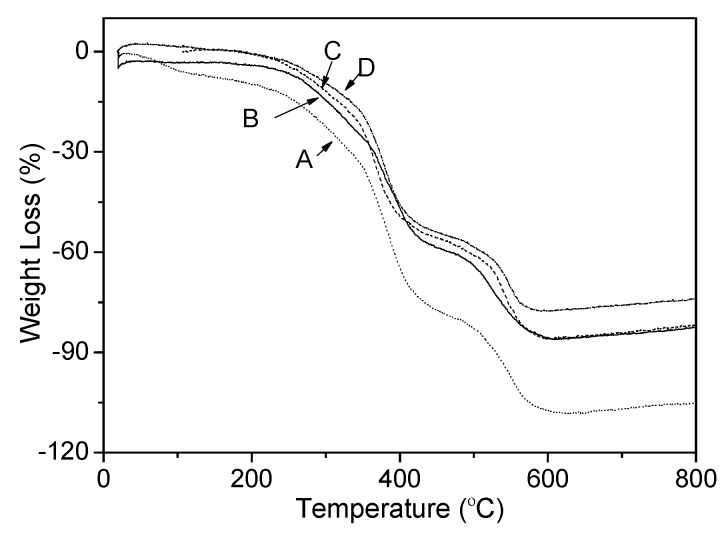
The TGA curves of GNS-based epoxy resin coating with different contents of (A) 0; (B) 0.1 wt %; (C) 0.4 wt %; and (D) 0.7 wt %.

The influence of PVP-rGO on the thermal stability of epoxy resin coating was characterized by TGA as shown in Figure 5. The TGA curves suggest that the thermal stability of epoxy resin coating could also be greatly improved by addition of PVP-rGO. For example, the temperature at 20% weight loss of neat epoxy resin coating was improved from 284.7 to 322.4 °C with the incorporation of 0.1 wt % PVP-rGO, and reached 356.3 °C in sample containing 0.7 wt % PVP-rGO, almost a 72 °C increase. The results were difficult to be observed in previous works [18,25,26,27]. The result was attributed to the physical barrier effect of the PVP-rGO that was brought about by its ultrahigh aspect ratio, which could create a “tortuous path” for the volatile degradation products. Moreover, the slow degradation of the polymer chains that absorbed at the matrix filler interface may also contribute to the greatly enhanced thermal stability of epoxy resin coating. In addition to this, this improvement in thermal stability could be attributed to the so called “tortuous path” effect of PVP-rGO, which delayed the escape of volatile degradation products and also char formation.

### 2.4. Protective Properties of GNS-Based Epoxy Resin Coating

**Figure 6:**
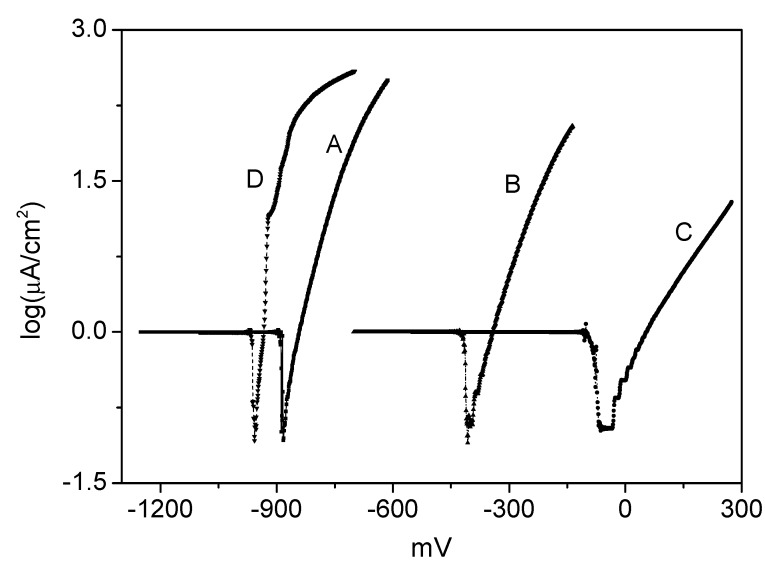
The Tafel plots for GNS-based epoxy resin coating with various content of (A) 0; (B) 0.1 wt %; (C) 0.4 wt %; and (D) 0.7 wt %.

The corrosion protection of Zn by a GNS-based epoxy resin coating was investigated by electrochemical impedance spectroscopy as shown in Figure 6. The corrosion potential (Ecorr), corrosion current (Icorr) and corrosion rate (Rcorr) were listed in Table 2. The Zn plate protected with GNS-based epoxy resin coating showed a lower Icorr and Rcorr than the neat epoxy resin coating, implying that the Zn plate protected with GNS-based epoxy resin coating was nobler toward electrochemical corrosion relative to the Zn plate protected with neat epoxy resin coating. For example, the GNS-based epoxy resin coating (0.7 wt %) showed a low Icorr of 0.18 μA/cm^2^, which is four times lower than the neat epoxy resin coating. The corresponding Rcorr of GNS-based epoxy resin coating (0.7 wt %) was *ca.* 0.3 mm/year, which was smaller than neat epoxy resin coating (1.3 mm/year). Although only a small proportion of PVP-rGO was incorporated into the epoxy resin matrixes, high corrosion protection was afforded. By comparison with that of epoxy resin coating containing others fillers in the previous works [28,29], the corresponding Rcorr of GNS-based epoxy resin coating was smaller. The good corrosion resistance may be attributed to the following two reasons: (1) epoxy could act as a physical barrier coating; (2) the well-dispersed graphene nanosheets embedded in epoxy matrix could prevent corrosion owing to a relatively higher aspect ratio than clay platelets, which enhances the oxygen barrier property of GNS-based epoxy resin coating [12].

**Table 2:** Anticorrosive performance of GNS-based epoxy resin coating as measured from electrochemical corrosion measurements.

Loading	Ecorr (mV)	Icorr (μA/cm^2^)	Rcorr (mm/year)
0	−900	0.75	1.3
1.0%	−47	0.69	1.2
4%	−408	0.48	0.85
7%	−957	0.18	0.3

**Figure 7:**
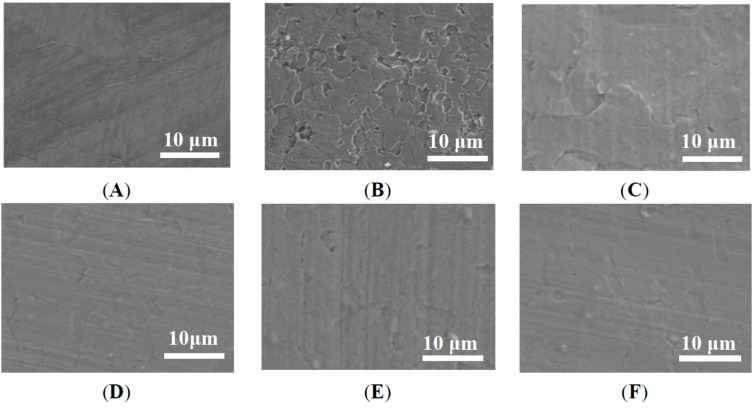
SEM images of uncoated Zn plate (**A**) before; and (**B**) after corrosion, Zn palate protected GNS-based epoxy resin coating with various content of (**C**) 0; (**D**) 0.1 wt %; (**E**) 0.4 wt %; and (**F**) 0.7 wt %.

Figure 7 showed typical SEM images of the corroded surface of uncoated and coated Zn plates after 6 days of immersion in 3.5 wt % NaCl solution. It clearly showed no distinct cracks or pitting corrosion for all coated Zn plates compared with the uncoated Zn specimen (in Figure 7B). Additionally, there were slight changes in the surface roughness of the coated specimen before and after corrosion (Figure 7A,C–F, respectively). These results indicate that epoxy resin coating is a good kind of protective layer for inhibiting the process of metal corrosion. Surface roughness of all coated specimens after corrosion was further compared as shown in Figure 7C–F. It was obvious that the surface roughness of Zn plate decreased with increases in PVP-rGO content, indicating the reduction in local corrosion. Based on the results of the electrochemical tests and corroded surface analysis, the GNS-based epoxy resin coating has higher doping content of PVP-rGO, it exhibited better corrosion resistance. The results were similar with the mechanical properties results being a function of PVP-rGO content. It is well-known that mechanical properties (such as Young’s modulus, and hardness) and erosion resistance play important roles in the corrosion behavior of materials: a low mechanical property with high porosity and erosion resistance allows for the penetration of solution, which leads to local corrosion [4,30]. The GNS-based epoxy resin coating (0.7 wt %) exhibited better mechanical properties and a finer and more dense surface as shown in Table 1 and Figure 2, respectively. In addition to this, graphene was a good inhibitor [2,3] and graphene distributed on epoxy resin matrix could form a good protective layer to act as a barrier to the penetration of solution. For these reasons, the GNS-based epoxy resin coating with good mechanical properties presented better corrosion resistance.

## 3. Experimental Section

### 3.1. Materials

Nature graphite flakes (325 mesh, 99.8%), sodium chloride (NaCl), epoxy resin and other chemicals and reagents used in this work are in analytical grade. Deionized water was used for preparation, dilution and analytical purpose.

### 3.2. Preparation of GNS-Based Epoxy Resin Coating

**Scheme 1:**
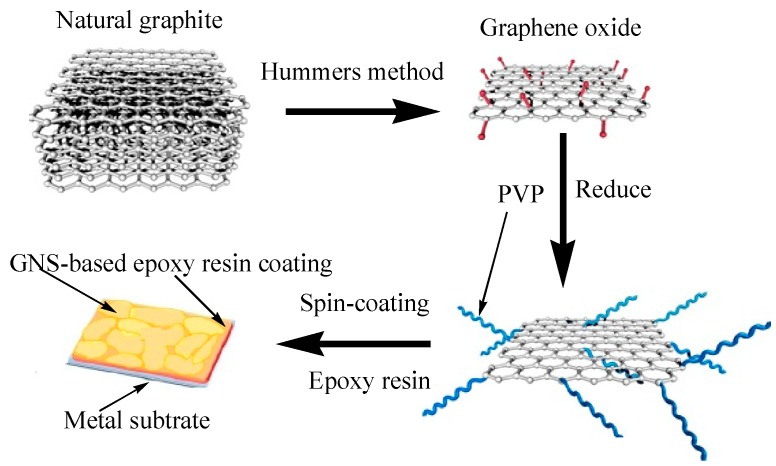
Synthetic process of GNS-based epoxy resin coating.

GNS-based epoxy resin coating was prepared as shown in Scheme 1. Firstly, Polyvinylpyrrolidone-stabilized graphene (PVP-rGO) was prepared from natural graphite by our group, and the preparation process was same as in previous work [15]. Secondly, an appropriate amount of PVP-rGO aqueous solution (2.5 mg/mL), calculated by 0, 0.1, 0.4 and 0.7 wt % with respect to epoxy resin, was introduced into the water-borne epoxy latex (34.6 wt %) and then further mixed under magnetic stirring for 1 h at room temperature. The resultant mixture was degassed and drop wisely cast onto a clean Zn plate and then cured at 80 °C for 2 h.

### 3.3. Mechanics Measurement of GNS-Based Epoxy Resin Coating by Nanoindentation

The nanoindentation tests were performed by using a Triboscope system (Histron Inc., Minneapolis, MN, USA). For all samples, this test was performed with a normal load of 800 mN according to the procedure described in ISO 14577 [16]. A Berkovich diamond indenter was used for performing indentation experiments. Prior to experiments, the tip area function was calibrated by using Oliver and Pharr methods [31] and a standard fused quartz sample. A typical loading hold-unloading sequence was used for indentation experiments. Then, in the unloading segment the tip was withdrawn from the sample surface at the same rate. For each sample, at least five indents were performed at different points on the surface. The Oliver and Pharr method was applied for analyzing the experiment data [31]. Eventually, in order to study the creep effect, the max load was kept constant for 25 min.

### 3.4. Corrosion Measurements

#### 3.4.1. Preparation of Sample Used in Corrosion Measurements

As a typical procedure to prepare sample-coated Zn for corrosion measurements, freshly prepared mixture solution containing PVP-rGO and epoxy resin were cast drop wisely onto the Zn plate (2 cm × 2 cm) and the polymer composite coatings dried in air for 2 h at 80 °C to give ~10 μm thick coatings, measured by a digimatic micrometer (Shanghai Tu Ming optical instrument Co., Ltd., Shanghai, China). The coated Zn plate were then mounted to the working electrode so that only the coated side of the Zn plate was in direct contact with the electrolyte. The other uncoated side and edges of the Zn plate were sealed with super-fast epoxy cement (SPARR).

#### 3.4.2. Electrochemical Measurements

All electrochemical measurements of corrosion potential and corrosion current were performed on a Volta Lab model 21 Potentiostat/Galvanostat in a standard corrosion test cell equipped with a saturated calomel reference electrode (SCE) and a working electrode, and all experimental data were repeated at least three times. The electrolyte was an aqueous solution containing 3.5 wt % sodium chloride. Open circuit potential (OCP) at the equilibrium state of the system was recorded as the corrosion potential [Ecorr (V)* vs.* SCE]. Corrosion current (Icorr) is determined by superimposing the straight line along the linear portion of the cathodic or anodic curve and extrapolating it through Ecorr. Corrosion rate (Rcorr, mm/year) is calculated from the following equation [32]:

(4) $$R_{corr}\left(mm/year\right)=\left(3270\times I_{corr}\times EW\right)/\text{ρ}$$

where *EW* is the equivalent weight (g), *I_corr_* is the corrosion current density (μA/cm^2^) and ρ is the density (g/cm^3^).

## 4. Conclusions

The GNS-based epoxy resin coating was prepared by a *situ* synthesis method, and the graphene modified by PVP could be well dispersed in the epoxy resin matrix and demonstrated good adhesion with epoxy resin matrix. A significant enhancement in the mechanical and thermal properties of epoxy resin coating was obtained with a 0.7 wt % loading of PVP-rGO, such as *ca.* 213% and 73 °C increase in Young’s modulus and thermal stability, respectively. At the same time, the plasticity index of epoxy resin coating decreased with increases in PVP-rGO content, in which the reduction of this parameter at 0.7 wt % loading was 26.9% compared with that of epoxy resin coating, indicating a significant enhancement in creep properties of GNS-based epoxy resin coating. Furthermore, erosion resistance of GNS-based epoxy resin coating was also investigated by the electrochemical measurements and immersion corrosion experiment in NaCl solution, respectively. It indicated that the GNS-based epoxy resin coating with higher mechanical and thermal properties had better erosion resistance. These results provide a novel route for improving mechanical and thermal properties of inhibited erosion polymer coating, which is expected to be used in thermal-mechanic-corrosion coupled environments.
